# The chemokine receptor CCR6 facilitates the onset of mammary neoplasia in the MMTV-PyMT mouse model via recruitment of tumor-promoting macrophages

**DOI:** 10.1186/s12943-015-0394-1

**Published:** 2015-06-06

**Authors:** Sarah T. Boyle, Jessica W. Faulkner, Shaun R. McColl, Marina Kochetkova

**Affiliations:** Department of Molecular and Cellular Biology, School of Biological Sciences, University of Adelaide, Adelaide, South Australia Australia; Centre for Molecular Pathology, University of Adelaide, Adelaide, South Australia Australia

**Keywords:** Breast Cancer, Mammary Gland, Chemokine Receptor, CCR6, Transgenic Mouse Model, Immune System, Macrophages

## Abstract

**Background:**

The expression of the chemokine receptor CCR6 has been previously correlated with higher grades and stages of breast cancer and decreased relapse-free survival. Also, its cognate chemokine ligand CCL20 has been reported to induce proliferation of cultured human breast epithelial cells.

**Methods:**

To establish if CCR6 plays a functional role in mammary tumorigenesis, a bigenic MMTV-PyMT CCR6-null mouse was generated and mammary tumor development was assessed. Levels of tumor-infiltrating immune cells within tumor-bearing mammary glands from MMTV-PyMT *Ccr6*^*WT*^ and *Ccr6*^*−/−*^ mice were also analyzed.

**Results:**

Deletion of CCR6 delayed tumor onset, significantly reduced the extent of initial hyperplastic outgrowth, and decreased tumor incidence in PyMT transgenic mice. CCR6 was then shown to promote the recruitment of pro-tumorigenic macrophages to the tumor site, facilitating the onset of neoplasia.

**Conclusions:**

This study delineated for the first time a role for CCR6 in the development of breast cancer, and demonstrated a critical function for this receptor in maintaining the pro-tumorigenic cancer microenvironment.

**Electronic supplementary material:**

The online version of this article (doi:10.1186/s12943-015-0394-1) contains supplementary material, which is available to authorized users.

## Introduction

Breast cancer is one of the leading causes of cancer-related death in women world-wide. Evasion of the immune system is a hallmark of cancer, and aids tumor cells to survive, intravasate, and potentially form distal metastases [1]. As such, the tumor microenvironment has a profound effect on the development and progression of malignancies, and it has been suggested that levels of infiltrating immune cells correlate with stage and aggressiveness of human breast cancer [2]. In particular, tumor-associated macrophages (TAMs) have been found to play an important part in facilitating breast tumor development [3] through polarization from a classically-activated “M1” anti-tumor resident cell within adult mammary tissue to an alternatively-activated “M2” pro-tumor phenotype [4]. This “switch” results in shifts in cell metabolism, a decrease in pro-inflammatory chemokine/cytokine production, poor antigen-presentation ability, and suppression of T cell responses. In addition, M2 TAMs promote angiogenesis, cell proliferation and tissue remodeling (reviewed in [5]).

Chemokines and their cognate receptors are involved in the development, migration and activation of many different types of immune cells, both adaptive and innate. Small molecular-weight proteins, chemokines bind to their cognate seven-transmembrane domain G-protein coupled receptors (GPCRs), activating a multitude of signaling pathways, which mediate many different homeostatic and inflammatory functions. Importantly, a large body of literature in the last decade has linked the action of chemokines and chemokine receptors to cancer progression and metastasis [6].

The CC-chemokine receptor CCR6 is expressed on dendritic cells [7, 8], regulatory T cells and various T helper lymphocyte subsets [9, 10], and mediates their migration and function via stimulation with its ligand CCL20 (also known as macrophage inflammatory protein (MIP)-3α [11]). CCR6 is also expressed on natural killer cells, B lymphocytes, neutrophils [12] and macrophages [10, 13]. Despite the significant role of TAMs in breast cancer, the expression and function of CCR6 within the macrophage population has not been shown within the mammary gland.

Interestingly, together with CCL20, CCR6 expression has been correlated with stage and prognosis in a variety of cancers including hepatocellular carcinoma [14, 15], colorectal carcinoma [16–18], glioma [19], and non-small cell lung cancer [20], and a function for CCR6 in regulation of cancer progression has been putatively demonstrated using cell lines and xenograft models [16, 18, 21, 22]. In breast cancer, higher CCR6 expression levels were linked with tumor stage and grade [23], and incidence of metastasis to the pleura [24]. Stimulation of *ex vivo* mammary peritumoral cells with CCL20 was found to increase their proliferation rate, invasiveness and migration [25]. CCL20 is also upregulated in human triple negative breast cancer cell lines [26]. Moreover, it was recently proposed that the presence of CCR6 may act as a prognostic factor for breast cancer patient survival [23]. However, no causative or functional link between the CCR6-CCL20 axis and progression of breast cancer has been documented to date.

In this study we have utilized a well-characterized transgenic model for breast cancer, in which the polyoma middle-T oncogene is activated under control of the mouse mammary tumor virus promoter (MMTV-PyMT) [27]. This transgenic model has been shown to closely mimic the stages of human breast disease from initial hyperplasia, through to ductal carcinoma *in situ* and invasive ductal carcinoma [28]. Crossing this transgenic mouse with a CCR6-null mouse to generate a bigenic MMTV-PyMT *Ccr6*^*−/−*^ animal model has allowed us to directly assess the role of CCR6 in mammary tumorigenesis *in vivo*. The results demonstrated that CCR6 promotes breast cancer initiation and progression through maintenance of pro-tumorigenic TAMs within tumor-bearing mammary glands, warranting further investigation of CCR6 as a possible therapeutic target.

## Results

### CCR6 expression increases throughout cancer development and results in a higher number of mammary tumors

**Fig. 1:**
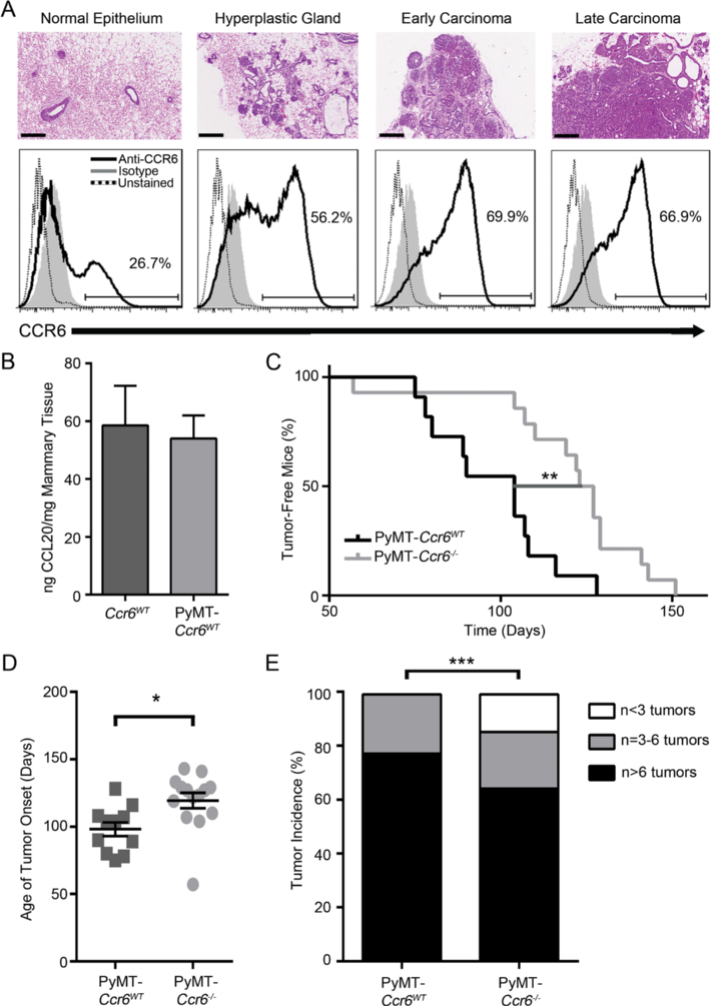
CCR6 expression increases throughout cancer development and results in a higher number of mammary tumors. **a** Top: Representative H&E images of mammary tissue from normal gland and various stages of PyMT-driven tumorigenesis as indicated. Scale bar is 200 μm. Bottom: Proportion of CCR6-positive epithelial cells (CD45-negative) purified from mammary glands at respective stages of tumorigenesis. 18 normal samples (2 glands/sample), 7 hyperplastic samples (2 glands/sample), 6 early carcinomas and 3 late carcinomas were analyzed. **b** ELISA for CCL20, the ligand for CCR6, in normal (left) and early neoplastic MMTV-PyMT *Ccr6* ^*WT*^ (right) mammary glands. n = 4 samples per genotype. **c** Kaplan-Meier analysis of the palpable tumor onset in MMTV-PyMT *Ccr6* ^*−/−*^ mice (n = 14) vs MMTV-PyMT *Ccr6* ^*WT*^ mice (n = 11). **d** Age of tumor onset in MMTV-PyMT *Ccr6* ^*−/−*^ mice and MMTV-PyMT *Ccr6* ^*WT*^ mice. **e** Mammary tumor incidence in MMTV-PyMT *Ccr6* ^*WT*^ (n = 9) and *Ccr6* ^*−/−*^ (n = 14) mice

To first determine whether CCR6 may play a role in the regulation of mammary neoplasia, we investigated expression of the receptor in CD45-negative normal mouse mammary cells, and cells from various tumor stages (representative H&E pictures in Fig. 1a). CCR6 was expressed on a low proportion of normal mammary cells, but this proportion was greatly amplified in accordance with increasingly higher grades of MMTV-PyMT cancer including initial hyperplasia, early carcinoma and late carcinoma as indicated (Fig. 1a). This is consistent with human breast cancer [23] and other mouse models of cancer [17]. Additionally, in both non-PyMT *Ccr6*^*WT*^ and MMTV-PyMT *Ccr6*^*WT*^ mammary tissues the ligand for CCR6, CCL20, was highly expressed at concentrations over 50 ng/mg tissue (Fig. 1b). These data raise the possibility of a role for CCR6 in breast cancer development.

To next establish the role of CCR6 deletion on mammary tumorigenesis, we compared the rate and total extent of PyMT-driven neoplasia between MMTV-PyMT *Ccr6*^*WT*^ and *Ccr6*^*−/−*^ mice. Tumor onset was significantly delayed in MMTV-PyMT *Ccr6*^*−/−*^ mice (Fig. 1c), with some mice not developing palpable tumors until 150 days old (21 weeks) compared to a maximum onset age of 130 days old (18 weeks) for MMTV-PyMT *Ccr6*^*WT*^ counterparts (Fig. 1d).

In order to assess the impact of CCR6 on the later stages of cancerogenesis, MMTV-PyMT *Ccr6*^*WT*^and *Ccr6*^*−/−*^ mice were sacrificed at 22–24 weeks of age and the total number of mammary tumors per mouse was determined. We found that MMTV-PyMT *Ccr6*^*−/−*^ mice had significantly decreased tumor incidence compared to MMTV-PyMT *Ccr6*^*WT*^ animals (Fig. 1e). Together, these results implicate CCR6 as being an important player in breast oncogenesis.

### CCR6 deletion significantly delays tumor initiation *in vivo*

**Fig. 2:**
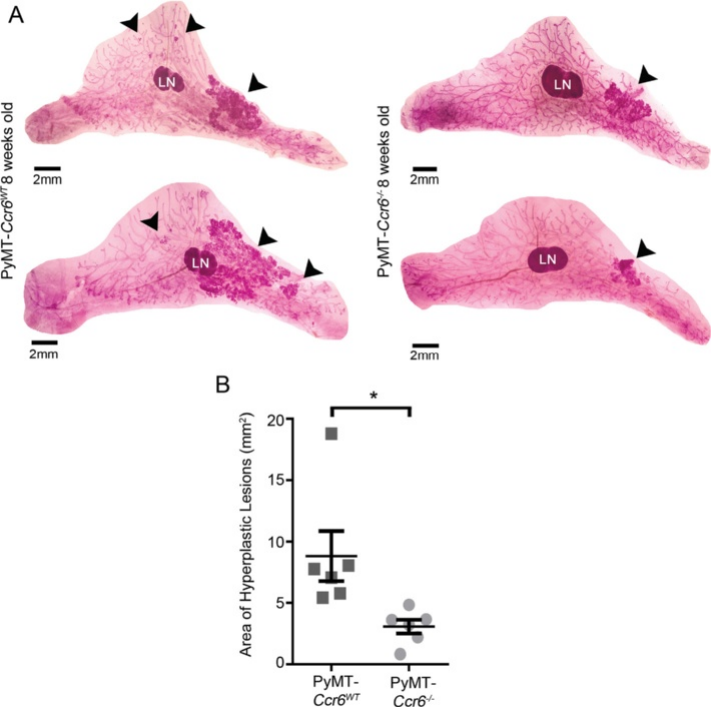
CCR6 deletion significantly delays tumor initiation *in vivo.* **a** Representative whole mount images of MMTV-PyMT *Ccr6* ^*WT*^ (n = 6) and *Ccr6* ^*−/−*^ (n = 6) mice at 8 weeks of age. LN = lymph node. Black arrowheads indicate hyperplastic lesions within the glands. **b** Quantitation of area of pre-neoplastic lesions in 8 week-old MMTV-PyMT mice

We then sought to examine whether CCR6 influenced early hyperplasia of mammary glands during tumor initiation as well as late stage tumorigenesis. Glands from 8-week-old MMTV-PyMT *Ccr6*^*−/−*^ and *Ccr6*^*WT*^ mice were extracted and whole mounted for quantitation of hyperplastic/early-neoplastic lesions (representative images from both genotypes shown in Fig. 2a). We found that the deletion of CCR6 significantly reduced the initial hyperplastic outgrowth within the gland (Fig. 2b), a common indicator of future breast cancer development. As the total area of PyMT-driven hyperplastic outgrowth per gland was reduced by threefold in CCR6-null animals, we concluded that the effect of CCR6 on mammary tumorigenesis is manifested very early on in cancer development.

The difference seen in early tumor initiation between MMTV-PyMT *Ccr6*^*WT*^ and *Ccr6*^*−/−*^ mice can potentially result from a difference in normal mammary development, which may then have translated into decreased hyperplasia. We therefore extracted pubertal mammary glands from non-PyMT 6-week-old *Ccr6*^*WT*^ and *Ccr6*^*−/−*^ mice. Representative glands are shown in Additional file 1: Figure S1a. When ductal epithelial growth was quantitated, we observed no statistically significant difference in ductal length, number of terminal end structures or branching between *Ccr6*^*WT*^ and *Ccr6*^*−/−*^ mice (Additional file 1: Figure S1b-d), and hence the overall effect of CCR6 deletion on normal mammary gland biology appears to be minimal and is unlikely to account for differences in PyMT-driven tumor development between the two genotypes. Furthermore, the levels of CCL20 were not statistically different between non-PyMT *Ccr6*^*WT*^ and MMTV-PyMT *Ccr6*^*WT*^ mammary tissues (Fig. 1b), demonstrating that the expression of CCL20 is not affected by the process of tumorigenesis. Taken together, these data show that early stage tumorigenesis is mediated by a CCR6-dependent mechanism, without affecting normal mammary morphogenesis.

### CCR6 promotes mammary gland neoplasia independently of cancer epithelial cells or stem-like cells

**Fig. 3:**
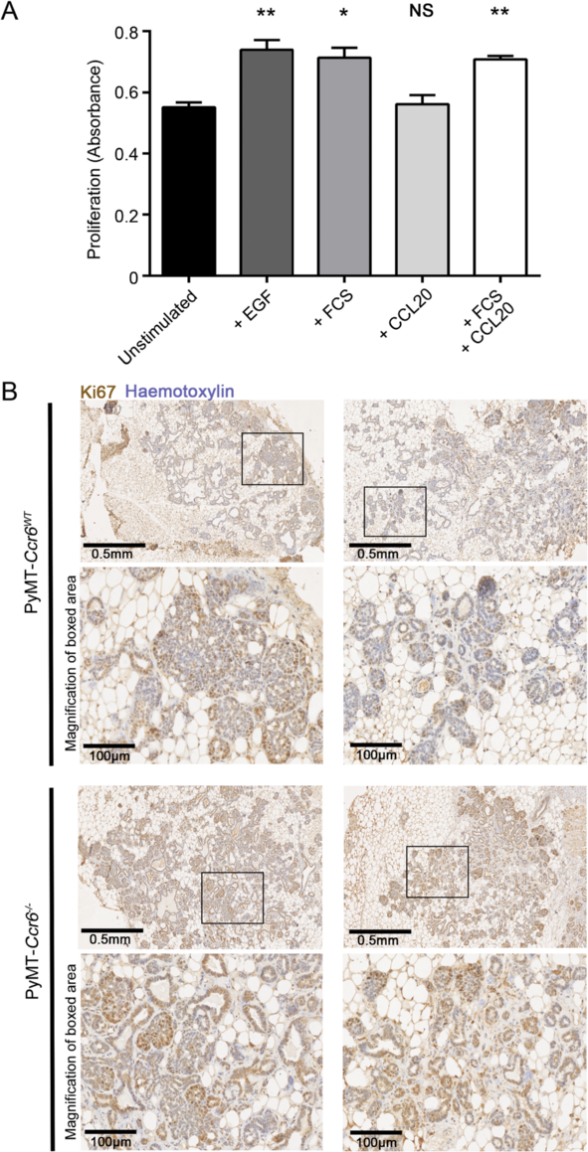
CCR6 promotes mammary gland neoplasia independently of cancer epithelial cells. **a** Proliferation assay of mammary epithelial cells purified from MMTV-PyMT *Ccr6* ^*WT*^ mice at the stage of early neoplasia with and without stimulation by recombinant CCL20 (100 ng/ml). Fetal calf serum (0.5 %) and EGF (20 ng/ml) were used as positive controls. Data are representative of 3 independent experiments, n = 3 mice per experiment. **b** Analysis of Ki67-positive proliferating cells within MMTV-PyMT *Ccr6* ^*WT*^ and *Ccr6* ^*−/−*^ mice at the stage of early neoplasia. Shown are representative fields from 2 separate tumors per genotype, displaying equal distribution of cells positive for Ki67

To investigate the mechanism underlying CCR6-driven mammary tumorigenesis, we studied the epithelial cell population to determine if CCR6 was having a direct effect on cell proliferation. Cells at the stage of early neoplasia from MMTV-PyMT *Ccr6*^*WT*^ mammary glands were assayed for proliferation upon stimulation with CCL20. No differences in cell proliferation were observed (Fig. 3a). Furthermore, Ki67 staining of sectioned hyperplastic mammary glands from MMTV-PyMT *Ccr6*^*WT*^ and *Ccr6*^*−/−*^ mice showed that epithelial cells in knock-out mice are still able to adequately proliferate and staining of Ki67 is equal to that in the *Ccr6*^*WT*^ (Fig. 3b). This suggests that the role of CCR6 in breast cancer is independent of epithelial cells.

We next determined whether CCR6 may exert its effect by skewing distinct cell populations within the bulk epithelium, as we have reported previously for the chemokine receptor CCR7 [29]. The current prevailing paradigm has mammary epithelial and breast cancer cells hierarchically organized with a self-renewing, quiescent, multipotent progenitor (or stem-like cell) population giving rise to basal and luminal progenitors which in turn differentiate into specific lineages making up the mammary gland and heterogenous breast tumors [30]. Recently, a number of immune mediators including chemokine receptors have been implicated in maintenance of the cancer stem-like cells within mammary tumors (reviewed in [31]). We therefore tested the potential link between the tumor-promoting function of CCR6 and breast cancer stem-like cell pools.

**Fig. 4:**
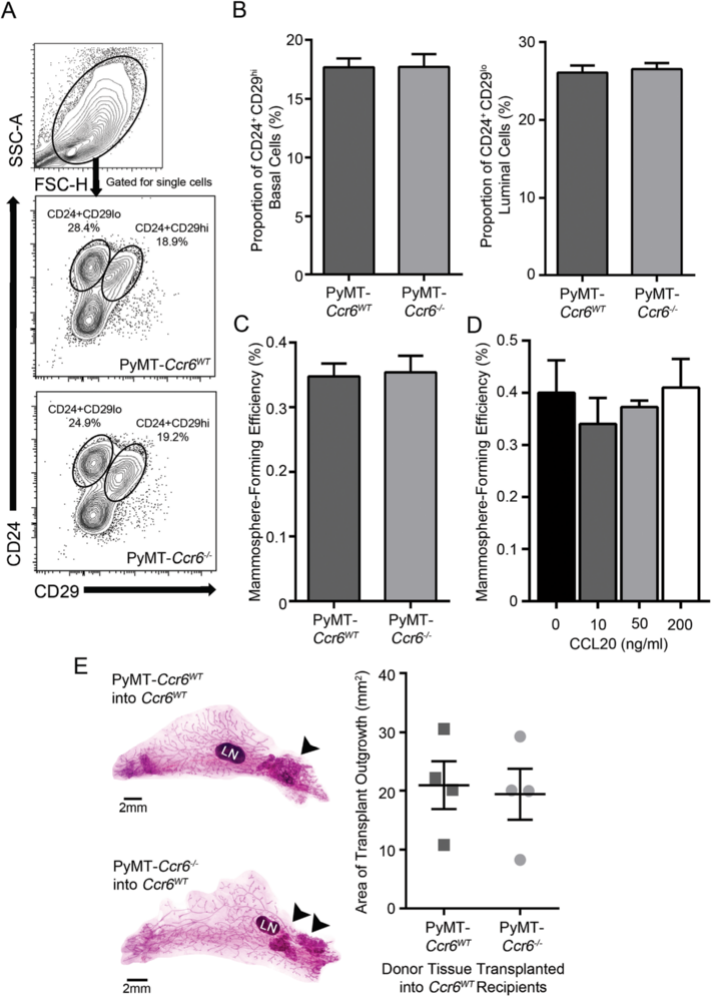
CCR6 promotes mammary gland neoplasia independently of cancer stem-like cells **a-b** Single cell suspensions (CD45-negative) from mammary glands with early neoplasia from MMTV-PyMT *Ccr6* ^*WT*^ and *Ccr6* ^*−/−*^ mice were analyzed by flow cytometry for the expression of CD24 and CD29 to quantitate the proportions of basal and luminal cell lineages. **c** Mammosphere-forming efficiency of cells isolated from MMTV-PyMT *Ccr6* ^*WT*^ and *Ccr6* ^*−/−*^ mammary glands. **a-c** Data are representative of 3 independent experiments, n = 6 mice per genotype per experiment. **d** Mammosphere-forming efficiency of MMTV-PyMT *Ccr6* ^*WT*^ cells stimulated with varying concentrations of CCL20. Data are representative of 3 independent experiments, n = 3 individual mice per experiment. **e** Representative whole mount images and quantitation of the neoplastic outgrowth area in *Ccr6* ^*WT*^ recipients of MMTV-PyMT *Ccr6* ^*WT*^ and MMTV-PyMT *Ccr6* ^*−/−*^ mammary tumor tissue transplants; n = 4 recipient mice per group, LN = lymph node. Black arrowheads indicate donor transplant outgrowth within the glands

Freshly isolated MMTV-PyMT *Ccr6*^*WT*^ and *Ccr6*^*−/−*^ mammary cells from pre-neoplastic mice at 8–9 weeks-old were assayed by flow cytometry for expression of cell surface markers CD24 and CD29 [32] (representative plots shown in Fig. 4a), which were previously used to define stem cells in the MMTV-PyMT [33, 34] and other breast cancer mouse models [35, 36]. We found that the deletion of CCR6 did not alter the proportions of the stem cell-enriched basal population (CD24^+^CD29^hi^) nor the luminal population (CD24^+^CD29^lo^) (Fig. 4b) in hyperplastic mammary glands.

We also investigated the effect of CCR6 ablation on functional stem-like mammary cancer cell pools using the mammosphere assay, which is used to select for colonies of early stem-like progenitors [37]. Pre-neoplastic MMTV-PyMT *Ccr6*^*WT*^ and *Ccr6*^*−/−*^ mammary cells were seeded into non-adherent mammosphere culture and allowed to grow for 7 days. The deletion of CCR6 did not alter mammosphere-forming efficiency (Fig. 4c), and when sphere cultures were stimulated with varying concentrations of CCL20, no change in their ability to form mammospheres (Fig. 4d) was observed, supporting results obtained using flow cytometric analysis.

Having found no difference in the proportion or function of stem cell-like pools within early tumorigenic lesions, we next tested a *bona fide* property of cancer stem cells – their ability to propagate tumors upon transplantation. MMTV-PyMT *Ccr6*^*WT*^ and *Ccr6*^*−/−*^ donor mammary tissue was transferred into recipient fat pads of syngeneic non-PyMT *Ccr6*^*WT*^ mice. Using this approach, we found no significant difference between MMTV-PyMT *Ccr6*^*WT*^ and *Ccr6*^*−/−*^ tissue in the ability to form outgrowths when transplanted into *Ccr6*^*WT*^ recipients (Fig. 4e), indicating that the deletion of CCR6 did not reduce the tumor-propagating capability of mammary epithelium.

Altogether, these results demonstrate that the role of CCR6 in breast cancer is independent of breast epithelial and progenitor cells, raising the possibility that its mechanism of action involves the tumor microenvironment.

### CCR6 mediates the recruitment of pro-tumorigenic macrophages to the mammary tumor microenvironment

**Fig. 5:**
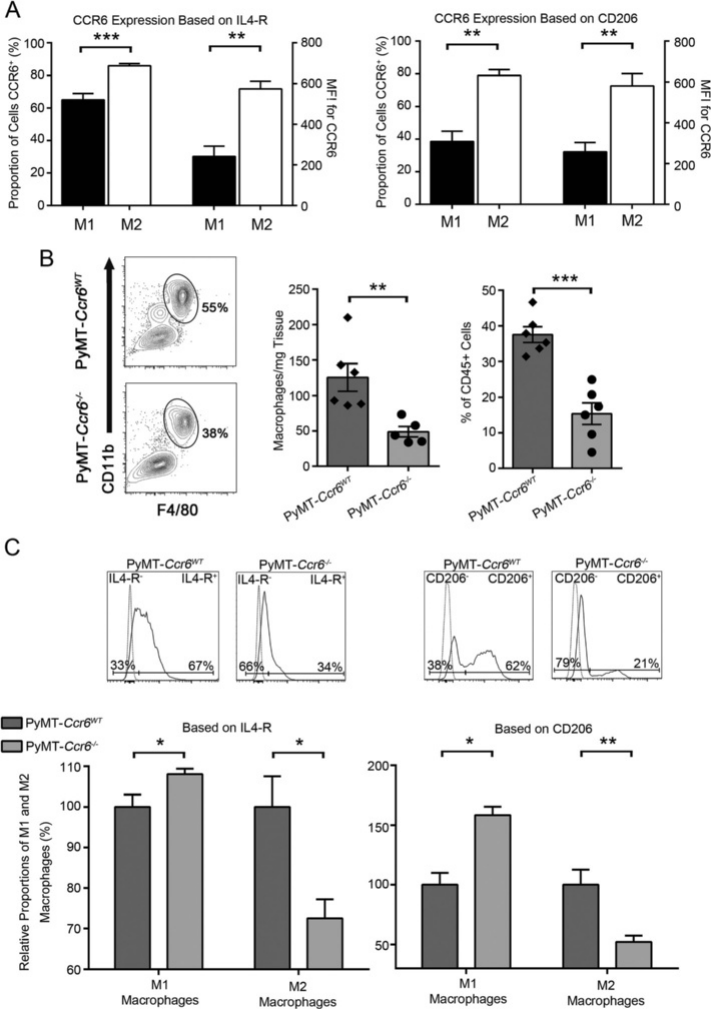
CCR6 mediates the recruitment of pro-tumorigenic macrophages to the mammary tumor microenvironment. **a** Comparison of proportions of CCR6-positive cells and levels of CCR6 within putative M1 and M2 macrophage populations, based on IL4-R (left) and CD206 (right) expression. MFI = mean fluorescence intensity. Representative results from 3 independent experiments. **b** Proportion of TAMs in mammary tumors from MMTV-PyMT *Ccr6* ^*WT*^ and *Ccr6* ^*−/−*^ mice as determined by flow cytometry. Data are representative of 3 independent experiments, n = 5-7 mice per genotype per experiment. **c** Proportions of putative M1 and M2 macrophage subtypes within the TAM population in mammary tumors from MMTV-PyMT *Ccr6* ^*−/−*^ mice relative to *Ccr6* ^*WT*^ *,* as determined by flow cytometry. Dotted line = FMO control. Data are representative of 4 independent experiments, n = 3 mice per genotype per experiment

To test whether the reduced mammary tumorigenesis caused by the deletion of CCR6 was due to an effect of the CCR6-CCL20 axis on the tumor microenvironment we next investigated by flow cytometry the levels and identity of tumor-infiltrating leukocytes in mammary tumors at the stage of early carcinoma (see Fig. 1a). Tumor-associated macrophages have been extensively implicated in tumor promotion both in the mammary gland and elsewhere, due to their role in angiogenesis, cell proliferation and tissue remodeling [5]. To initially examine the polarization of TAMs in the MMTV-PyMT mice, macrophages were assessed for expression of prototypic markers interleukin-4-receptor (IL4-R) and mannose receptor (CD206), which have been used previously in flow cytometric analysis to distinguish alternatively-activated M2 macrophages from classically-activated M1 [38–42]. We estimated using these markers that a high proportion of TAMs were of an M2-like phenotype (Additional file 1: Figure S2a), as has been suggested previously for MMTV-PyMT mammary tumors [43]. Interestingly, CCR6 was found to be highly expressed within the TAM population as it was detected on greater than 60 % of total macrophages (Additional file 1: Figure S2b). Most importantly, CCR6 was expressed at higher levels and on a significantly higher proportion of putative M2 macrophages (up to 90 %) than M1 (Fig. 5a and Additional file 1: Figure S2b), using both IL4-R and CD206 to delineate the populations. This strong correlation potentially implicates CCR6 in the regulation of pro-tumorigenic macrophages within the mammary gland microenvironment.

We then assessed the levels of macrophages in mammary tumors from MMTV-PyMT *Ccr6*^*WT*^ and *Ccr6*^*−/−*^ mice, and found that the proportion and overall numbers of TAMs were significantly reduced in MMTV-PyMT *Ccr6*^*−/−*^ mammary tumors relative to *Ccr6*^*WT*^ (Fig. 5b). Furthermore, the deletion of CCR6 resulted in a shift towards an M1 macrophage phenotype, defined by both IL4-R and CD206 prototypic markers (Fig. 5c).

The deletion of CCR6 also resulted in reduced trafficking of dendritic cells to the tumor site, consistent with previous studies, which demonstrated a reduced overall migratory ability of dendritic cells in CCR6-null mice [44, 45]. Although, overall numbers of tumor-infiltrating dendritic cells were much lower than the corresponding macrophage population. Notably, the majority of tumor-infiltrating dendritic cells were CCR6-positive, consistent with previous findings [12] (Additional file 1: Figure S3). We have also assessed CCR6 expression on B cells and specific T cell subsets (helper T cells (Th), cytotoxic T cells (Tc), and regulatory T cells (Treg)) within mammary tumors. In agreement with earlier reports for various biological settings [12, 46], all tested infiltrating leukocyte subsets expressed CCR6 at varying levels. When CCR6 was ablated, only the Tc cell subset showed a slight increase in the MMTV-PyMT *Ccr6*^*−/−*^ as a proportion of CD45^+^ tumor-infiltrating cells, however no significant differences were found in total cell numbers between the two genotypes (Additional file 1: Figures S4 and S5).

Our findings thus suggest that CCR6 promotes mammary tumorigenesis through an epithelium-independent mechanism involving tumor-infiltrating macrophages.

### CCR6-mediated pro-tumorigenic macrophages promote breast cancer *in vivo*

**Fig. 6:**
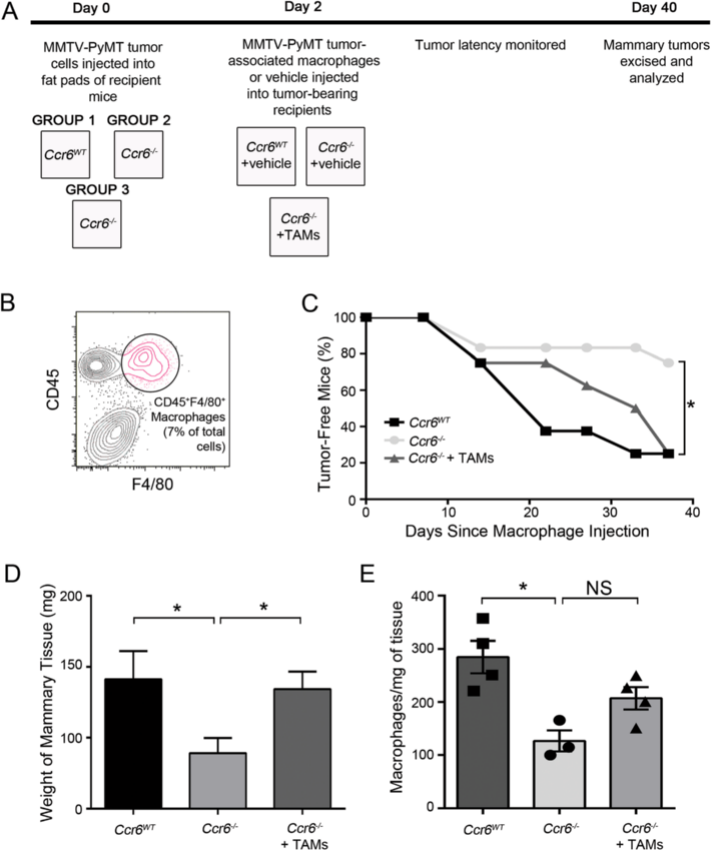
CCR6-mediated pro-tumorigenic macrophages promote breast cancer *in vivo*. **a** Schematic of macrophage reconstitution assay to determine contribution of macrophages to mammary tumorigenesis in the context of CCR6 deletion. TAM = tumor-associated macrophage. **b** FACS plot showing the sorted macrophage population used for reconstitution. **c** Tumor-free survival curves over the course of the experiment. **d** Tumor weight of control tumors generated in *Ccr6* ^*WT*^ recipients and in *Ccr6* ^*−/−*^ recipients ± TAMs. n = 6-8 tumors per group. **e** Macrophage numbers in experimental groups at end-point, as assessed by flow cytometry. n = 3-4 samples per group

We then sought to provide definitive evidence for the macrophage-mediating function of CCR6 in mammary tumor promotion using an *in vivo* macrophage reconstitution assay. Reconstitution assays, sometimes referred to as “add-back” assays, are frequently used to underscore a role for various cellular subsets in multiple pathological settings, and macrophage reconstitution has been used previously in mammary gland studies [47]. A schematic of the experimental setup is shown in Fig. 6a. MMTV-PyMT mammary tumor cells from *Ccr6*^*WT*^ donor mice were purified and transplanted into the inguinal mammary fat pads of non-PyMT *Ccr6*^*WT*^ and *Ccr6*^*−/−*^ recipients at 5 weeks of age. Two days post-transplantation, TAMs (CD45^+^F4/80^+^) were sorted from excised and dissociated MMTV-PyMT *Ccr6*^*WT*^ tumors (Fig. 6b) and orthotopically injected into a group of *Ccr6*^*−/−*^ recipients as specified in Fig. 6a. All other recipients received sham injections with vehicle only, followed by assessment of mammary tumor growth 6 weeks later.

In agreement with the results from spontaneous tumorigenesis studies, we have found that tumors grew significantly slower in the *Ccr6*^*−/−*^ hosts compared to *Ccr6*^*WT*^, indicating that CCR6 is required in the mammary stroma for robust tumor development. However, when the reduced macrophage phenotype was restored in *Ccr6*^*−/−*^ mice through orthotopical injections, the tumor latency was significantly shortened, approaching that of the *Ccr6*^*WT*^ mice (Fig. 6c).

It was further found that the supplementation of *Ccr6*^*−/−*^ mice with TAMs restored the efficiency of tumor growth (measured by weight of tumor-bearing mammary glands) within these mice to that seen in the *Ccr6*^*WT*^, whilst *Ccr6*^*−/−*^ mice that received sham injections displayed reduced tumorigenesis (Fig. 6d) as seen in the spontaneous model (see Fig. 1).

Enumeration of macrophages within grafted tumors in *Ccr6*^*WT*^ and *Ccr6*^*−/−*^ mice (Fig. 6e) paralleled the results seen in spontaneous mammary tumors (see Fig. 5b). Whilst there was an upward trend towards increased TAMs in the *Ccr6*^*−/−*^ mice that received TAM injections, the difference was not statistically significant (Fig. 6e). This, combined with the decreased tumor growth in the CCR6-null mice, indicated that the support of macrophages is essential at the early stages of tumor growth.

We have thus established an essential role for CCR6 in the tumor microenvironment, providing a causative link between this receptor, infiltrating macrophages and mammary tumor development. Hence, therapeutic opportunities may be explored to control breast cancer progression, via manipulation of the CCR6-CCL20 axis to control tumor-promoting macrophages.

## Discussion

We show here that the deletion of the chemokine receptor CCR6 caused a delay in tumor onset and decreased mammary tumor incidence *in vivo* in the MMTV-PyMT transgenic mouse model. We have determined that the underlying basis of the CCR6 oncogenic function is the increase in numbers of infiltrating pro-tumorigenic macrophages.

Multiple functional roles have been suggested for members of the chemokine family and their receptors in breast cancer pathophysiology [6], however little data using animal models is available to support these observations. The expression of CCR6 has been reported to correlate with higher stage and grade of human breast cancer, and has been proposed as a prognostic tool for determining relapse-free survival in breast cancer patients [23]. However, a causative link *in vivo* has yet to be demonstrated. We have employed the well-characterized MMTV-PyMT transgenic mouse model of breast cancer, and have found that CCR6 facilitates an earlier tumor onset and an increased incidence of mammary tumors. Of note, CCR6 affects mammary tumorigenesis from as early as the hyperplastic, or hyper-proliferative, stage. This initial phase of tumor development remains largely uncharacterized, despite being the most treatment-effective stage of cancer progression. Therefore, a better understanding of tumor initiation is crucial in order to develop therapies that target the tumorigenic process at the early stages of breast cancer.

When CCR6 was deleted in the MMTV-PyMT mouse, tumor latency was significantly extended, and these mice developed fewer mammary tumors than their *Ccr6*^*WT*^ counterparts. However, CCR6 deletion did not affect tumorigenic properties of the epithelium as we have found with the chemokine receptor CCR7 [29]. Stimulation with CCL20 did not result in an increased proliferation rate of purified mammary epithelial cells from hyperplastic glands or tumorous lesions in contrast to previous studies with primary human breast peritumoral cells [25]. Furthermore, the deletion of CCR6 did not lead to decreased numbers of Ki67-positive proliferating cells within intact tumor-bearing mammary glands, pointing to an epithelial-independent function of this receptor in breast cancer.

We have also observed that the loss of CCR6 did not alter the numbers and functional properties of mammary cancer stem-like cells. Transplantation experiments in particular demonstrated that the presence of CCR6 in donor epithelium was not required for tumor propagation in recipient mammary glands.

Further investigation demonstrated that CCR6 functions via organization of the immune system during the early stage of mammary carcinogenesis. We have shown that the levels of TAMs are reduced by almost threefold when CCR6 is deleted. TAMs, which have been previously identified in MMTV-PyMT tumors [48], are widely reported to support the development of cancer [3, 49] and in the tumor microenvironment they are generally thought to polarize towards an alternatively-activated M2 pro-tumor phenotype relative to the classic M1 anti-tumor phenotype [4]. Whilst the TAMs in MMTV-PyMT tumors are polarized towards an M2-like subtype, we have shown that the presence of CCR6 maintains M2 TAMs as the predominant phenotype. Therefore, it is plausible to suggest that CCR6 in breast cancer functions to recruit pro-tumorigenic macrophages to the tumor immuniche [31], to support growth of transformed epithelial cells and cancer stem cells, as TAMs in the MMTV-PyMT model have also been shown to also maintain stem-like cells [50].

CCR6 is not expressed on peripheral blood monocytes, and is thought to only be acquired upon their differentiation into macrophages, induced by the tumor microenvironment [12]. In accordance with this, we found that a high proportion of macrophages within PyMT-driven mammary tumors express CCR6, which has not been previously demonstrated in breast cancer. Also of potential importance is the fact that up to 90 % of pro-tumorigenic M2-like TAMs expressed CCR6. Our findings parallel results from a recent study which showed that CCR6-null mice bearing the adenomatosis polyposis coli (APC)^min^ transgene (a well-characterized model for gastrointestinal tumorigenesis) developed fewer intestinal adenomas and polyps, and that the effect of CCR6 was also linked to a significant reduction in F4/80^+^ macrophages [17]. Interestingly, Liu *et al.* also recently demonstrated that the ligand CCL20 is secreted from both macrophages and tumor cells in another mouse model of colorectal cancer, potentially suggesting common regulatory mechanisms and a universal role for CCR6 in tumors of various etiology [51].

MMTV-PyMT cancer cell transplant experiments showed that tumor growth in a CCR6-null microenvironment was significantly inhibited compared to wild-type microenvironment conditions, directly demonstrating that the mammary stroma is dependent upon CCR6 for adequate tumor initiation and growth support. The reconstitution of this CCR6-negative microenvironment with MMTV-PyMT *Ccr6*^*WT*^ TAMs restored the tumor-promoting properties of mammary stroma, indicating that breast cancer can be therapeutically targeted through manipulation of the CCR6-CCL20 axis to control tumor-infiltrating macrophages.

CCR6 deletion has also impeded recruitment of dendritic cells into PyMT-driven mammary tumors. Recruitment of dendritic cells into various solid tumors has been well-documented (reviewed in [52]), and their role in tumor progression is mainly centered around tumor antigen presentation to lymphocyte subsets leading to anti-tumor immune responses [53, 54]. Furthermore, there is some evidence supporting direct tumoricidal activity of dendritic cells [53]. As previous studies have reported an intrinsic requirement for CCR6 in migration and fundamental functions of dendritic cells [44, 45], our finding of the reduced infiltration of dendritic cells in mammary tumors may not be a facet of cancer development in MMTV-PyMT *Ccr6*^*−/−*^ mice, but is an inherent property of dendritic cell migration at a slower rate after CCR6 deletion.

## Conclusions

In conclusion, we show here that CCR6 plays a significant role in the initiation and at the early stage of breast cancer development *in vivo* by mediating recruitment of pro-tumorigenic macrophages to the tumor site, and thus facilitating further progression to advanced stages of mammary neoplasia. Results presented in this study therefore suggest CCR6 as a potential target for therapeutic intervention in early breast cancer.

## Methods

### Mice

Mice were maintained in pathogen-free conditions in the University of Adelaide’s Laboratory Animal Services facility. *Ccr6*^*−/−*^ mice have been described previously [44]. *Ccr6*^*−/−*^ females were crossed with C57BL/6 MMTV-PyMT males and the heterozygous offspring were interbred to produce MMTV-PyMT *Ccr6*^*WT*^ (wild-type for CCR6) and bigenic MMTV-PyMT *Ccr6*^−/−^ mice on the C57Bl/6 background. The University of Adelaide institutional animal ethics committee approved all animal experimental protocols.

### Histology

Mouse mammary tissues were extracted, fixed in formalin and embedded in paraffin before sectioning at 5 μm. Haemotoxylin and eosin staining was carried out according to standard protocols. For immunohistochemical analysis of Ki67, slides were immersed in 0.5 % hydrogen peroxide in methanol for 10 minutes to inhibit endogenous peroxidase activity, followed by antigen retrieval by boiling slides in 0.1 M sodium citrate buffer under pressure. Slides were blocked for 20 minutes in 5 % normal rabbit serum in TBS/0.1 % Tween to prevent non-specific antibody binding, and then incubated overnight at 4 °C with mouse anti-Ki67 antibody (Vector Labs) according to the manufacturer’s instructions. Specific antibody binding was detected using the EnVision Dual Link System (Vector Labs), followed by incubation with diaminobenzidine (DAB) substrate (Dako). Sections were counterstained with haemotoxylin, dehydrated and mounted.

### Enzyme-linked immunosorbent assay

Wells were coated with anti-CCL20 capture antibody (R&D Systems) at 2 μg/ml overnight followed by a blocking step in PBS/3%BSA. Homogenized mammary tissue lysates (in PBS containing 10 % glycerol and 1x protease inhibitor) were added for 1.5 hours at 37 °C. Biotinylated anti-CCL20 detection antibody (R&D Systems) was added at 50 ng/ml for 1 hour at 37 °C followed by incubation with streptavidin-HRP (Rockland) for 30 minutes at room temperature. Wells were washed with PBS/0.05 % Tween after each incubation.

### Whole mount staining

Mammary glands were mounted on slides, fixed in Carnoy’s (30 % glacial acetic acid, 30 % absolute ethanol, 10 % chloroform), stained overnight in Carmine Alum (Stem Cell Technologies), then dehydrated and mounted using Permount (ThermoFisher Scientific). Image “stitching” and analysis were performed using Image J software.

### Processing mouse mammary tissue to single cell suspension

Mouse mammary gland/tumor tissue was minced and then digested in Dulbecco’s Modified Eagle Medium (DMEM, Gibco) containing 1 mg/mL collagenase III, 100U/mL hyaluronidase (both from Worthington), 2 % fetal calf serum (FCS) and penicillin-streptomycin for 3–4 hours with gentle tilting. Organoids were further digested for 15 minutes with 6U/mL dispase (Gibco) in PBS and 20U/mL DNase I (Merck), and red blood cells were lysed by isotonic lysis buffer (150 mM NH_4_Cl in 17 mM Tris–HCl, pH 7.2). Single cells were obtained by filtration through a 70 μm nylon mesh.

### Proliferation assay

Isolated mouse mammary cells were plated in adherent culture (1:1 mixture of DMEM and Ham’s F12 medium (Gibco) with 10 % FCS, supplemented with 20 ng/ml EGF, 5 μg/ml insulin, 0.5 μg/ml hydrocortisone, penicillin-streptomycin, and 0.25 μg/ml fungazone) in a 96-well plate. The following day the medium was replaced by DMEM with no additives, and after 2 hours of starvation cells were stimulated with FCS (0.5 %) ± CCL20 (a gift from the late Professor Ian Clark-Lewis) at a concentration of 100 ng/ml. Stimulation with EGF at 20 ng/ml was used as a positive control. The cell proliferation assay was carried out 24 hours later using the XTT Cell Proliferation Kit (ATCC) according to manufacturer’s instructions.

### Flow cytometry

Single cell suspensions from processed mammary glands were incubated for 30 minutes on ice in PBS/0.5%BSA with anti-mouse primary antibodies to cell surface markers as indicated. Antibodies used were as follows: PE-conjugated anti-CCR6 (R&D), AlexaFluor647-conjugated anti-CCR6, PerCP/Cy5.5-conjugated anti-CD11c, PerCP/Cy5.5-conjugated anti-CD206, FITC-conjugated anti-CD29 (all from BioLegend), BV421-conjugated anti-B220, PE/Cy7-conjugated anti-CD11b, PE-conjugated anti-CD24, FITC-conjugated anti-CD4, APC-conjugated anti-CD45, biotinylated anti-CD45.2, FITC-conjugated anti-CD45.2, BV510-conjugated anti-CD8a, PE-conjugated anti-IL4-R (all from BD Biosciences), PE/Cy7-conjugated anti-CD3e, FITC-conjugated anti-F4/80 (both from eBioscience), and biotinylated anti-F4/80 (Life Technologies). When required, cells were also permeabilized using the FoxP3 Staining Kit, and incubated with PerCP/Cy5.5-conjugated anti-FoxP3 (both from eBioscience).

Samples containing biotinylated antibodies were further stained with BV510-conjugated streptavidin (BD Biosciences) in PBS/0.5 % BSA for 30 minutes. Fluorescence-minus-one (FMO) samples or cells stained with conjugated isotype control antibodies only were used as negative controls. After staining cells were fixed in 1 % paraformaldehyde and flow cytometry carried out using FACSCanto or LSRII equipment (BD). Data analysis was performed using FlowJo software (Tree Star Inc.). All flow cytometry data presented has been gated to exclude dead cells and debris using FSC-A/SSC-A, and to exclude doublets using FSC-A/FSC-H plots.

### Mammosphere assay

Freshly isolated mammary cells were seeded into ultra-low attachment plates (Corning Inc.) at a concentration of 4x10^4^/ml, in a 1:1 mixture of DMEM and Ham’s F12 medium supplemented with 1xB27 (Invitrogen), 20 ng/ml FGF, 20 ng/ml EGF, 4 μg/ml heparin (Sigma Aldrich), penicillin-streptomycin and 0.25 μg/ml fungazone. Mammosphere cultures were incubated at 37 °C for 7 days ± CCL20 at varying concentrations before manual enumeration under a light microscope.

### Mammary Fat Pad transplants

Mammary gland fragments of 1 mm^3^ size from donor MMTV-PyMT mice (18 weeks-old) were transplanted into contralateral sides of anaesthetized congenic non-PyMT recipient mice as indicated (8 weeks-old) within the inguinal mammary glands, and were monitored for adverse reactions to surgery. After 7 weeks, recipient glands were extracted and whole mounted for quantification.

### Macrophage reconstitution assay

Mammary tumor cell suspensions were prepared from MMTV-PyMT *Ccr6*^*WT*^ mice at 15 weeks-old as described above and injected into the fourth inguinal mammary fat pads of anaesthetized 5 week-old *Ccr6*^*WT*^ and *Ccr6*^*−/−*^ recipients in 80:20 % DMEM:Matrigel (BD), at 100,000 cells/gland.

Two days later, tumor-associated macrophages were sorted from MMTV-PyMT *Ccr6*^*WT*^ excised and dissociated mammary tumors based on CD45^+^F4/80^+^ expression. 50,000 TAMs per gland were injected in DMEM orthotopically into the inguinal glands of *Ccr6*^*−/−*^ tumor cell recipients. Control groups of *Ccr6*^*−/−*^ and *Ccr6*^*WT*^ tumor cell recipients were sham-injected with vehicle only.

Tumor development was monitored for 6 weeks, then mice were sacrificed and tumors extracted for analysis.

### Statistical analysis

Analyses were carried out using GraphPad Prism and data is presented as mean ± SEM unless otherwise indicated. Significant statistical difference was estimated using student’s t-tests, ANOVA for multiple comparisons, or chi-square tests for distribution analysis. Tumor-free survival curves for spontaneous tumors were graphed using the Kaplan-Meier method and compared by the log-rank statistic (Mantel-Cox test). Tumor-free survival curves for the reconstitution assay were compared using 2-way ANOVA with Tukey’s multiple comparison test. P-values were used to denote statistical significance. Levels of significance were *p ≤ 0.05, **p ≤ 0.01, and ***p ≤ 0.001.

## Additional file

Additional file 1: Figures S1-S5. Accompanies the manuscript.

## Abbreviations

FMO: Fluorescence-minus-one
GPCR: G-Protein coupled receptor
IL4-R: Interleukin-4-receptor
MFI: Mean fluorescence intensity
MIP-3α: Macrophage inflammatory protein-3α
MMTV: Mouse mammary tumor virus
PyMT: Polyoma middle-T
TAM: Tumor-associated macrophage
Tc: Cytotoxic T cell
Th: Helper T cell
Treg: Regulatory T cell
WT: Wild-type
