# Supplementary material for: The chemokine receptor CCR6 facilitates the onset of mammary neoplasia in the MMTV-PyMT mouse model via recruitment of tumor-promoting macrophages
Source: Mol Cancer. 2015 Jun 6;14:115. doi: 10.1186/s12943-015-0394-1 (PMC4464622; doi:10.1186/s12943-015-0394-1)
Supplement: Additional file 1: Figures S1-S5. — Accompanies the manuscript. [file 12943_2015_394_MOESM1_ESM.pdf]

Boyle, ST *et al*. The chemokine receptor CCR6 facilitates the onset of mammary neoplasia in the MMTV-PyMT mouse model via recruitment of tumor-promoting macrophages.

Additional File 1:
Supplementary Figures S1-S5

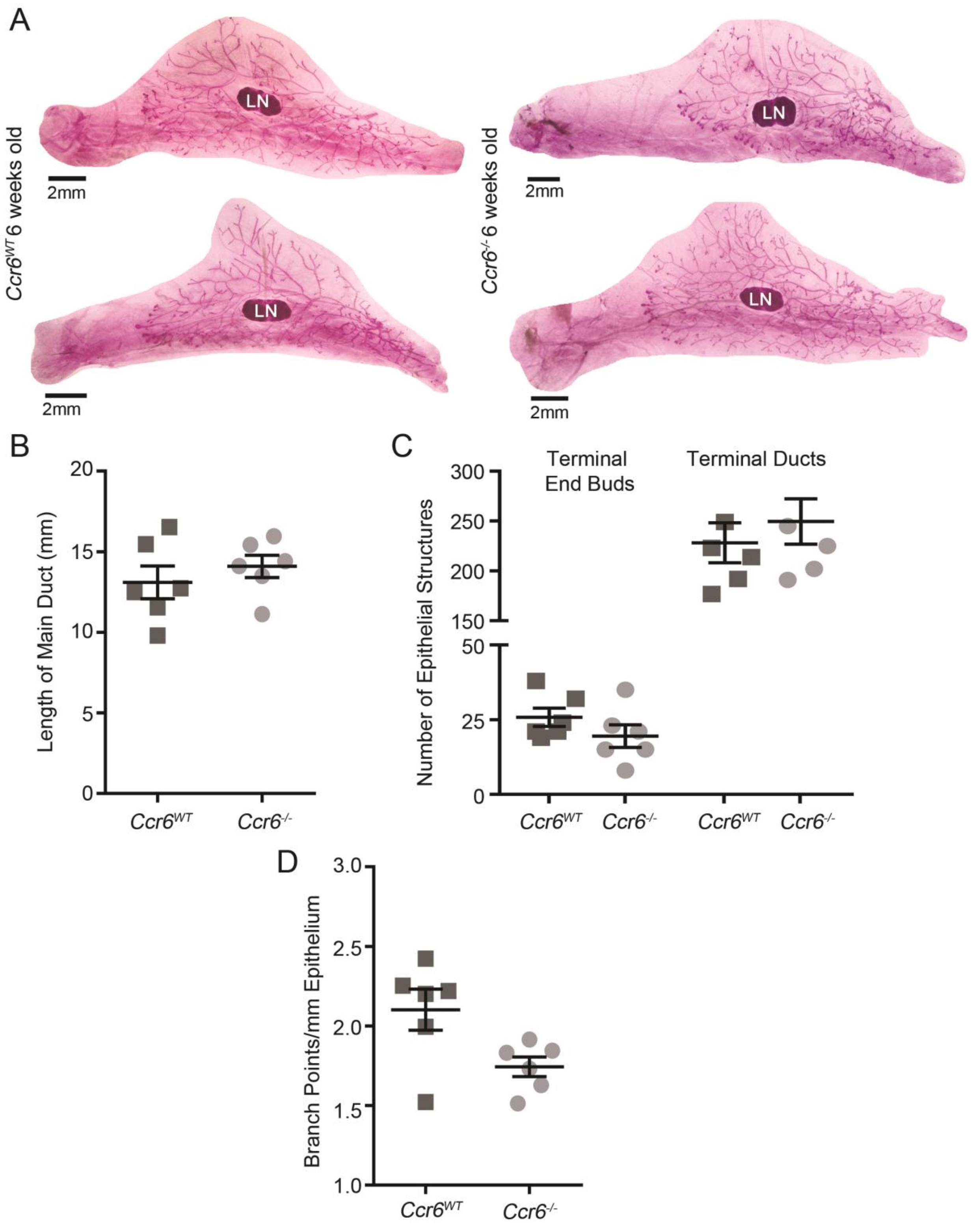

Figure S1: The role of CCR6 in normal mammary development

(a) Representative whole mount images of *Ccr6*^{WT} (n=6) and *Ccr6*$^{-/-}$ (n=6) mice at 6 weeks of age showing development of mammary epithelium. LN=lymph node. **(b-d)** Quantitation of epithelial growth, by length of main duct **(b)**, number of end structures **(c)** and branching **(d)**.

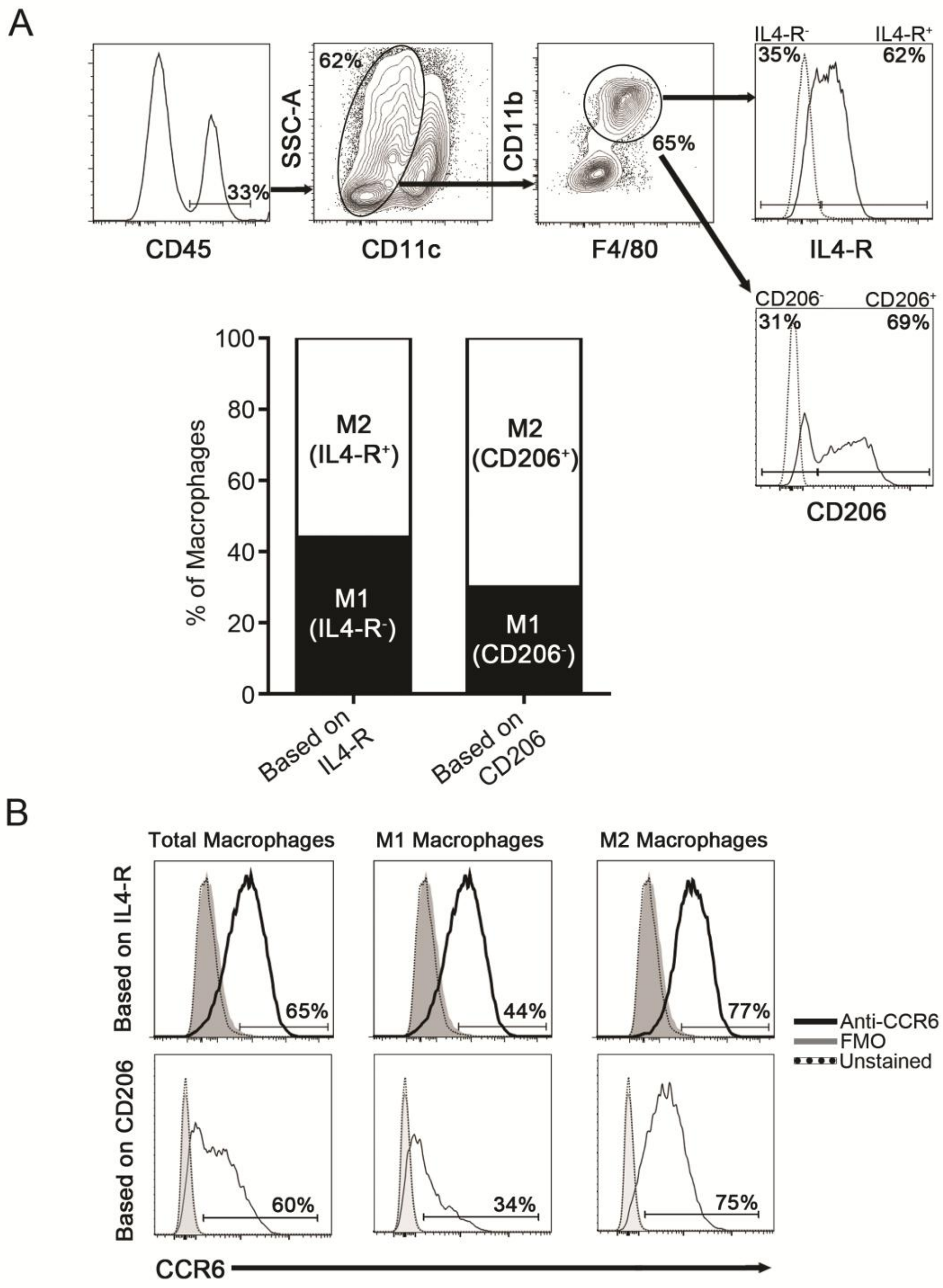

Figure S2: Analysis of infiltrating macrophages in MMTV-PyMT mammary tumors

(a) Analysis of M1 anti-tumor and M2 pro-tumor macrophages within the total macrophage population ($CD45^+CD11c^-CD11b^+F4/80^+$), based on IL4-R expression and CD206 expression as indicated, where M1 macrophages are classed as IL4-R or CD206-negative and M2 are IL4-R or CD206-positive. Dotted line=FMO control. M1 and M2 cell proportions (bottom) are presented as percentage of the total macrophage population. **(b)** Representative flow cytometry plots of CCR6 expression within the total, M1 and M2 macrophage populations as based on IL4-R and CD206 (gated as in (a)). **(a-b)** Representative results from 3 independent experiments.

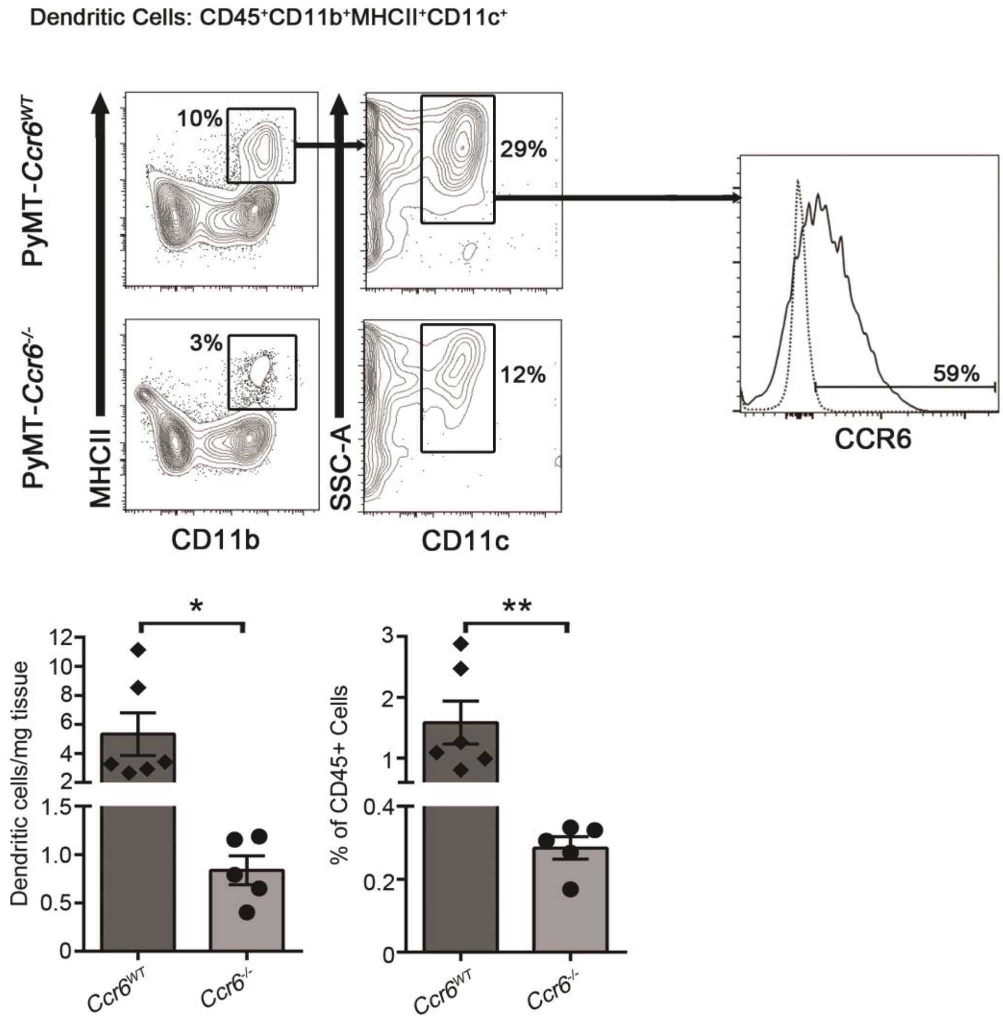

Figure S3: Infiltrating dendritic cells in MMTV-PyMT *Ccr6*^{WT} and *Ccr6*$^{-/-}$ mammary tumors as determined by flow cytometry

All cells were gated for CD45^{+}. MMTV-PyMT *Ccr6*^{WT} dendritic cells were assessed for CCR6 expression (right), and proportions and numbers of dendritic cells compared between MMTV-PyMT *Ccr6*^{WT} and *Ccr6*$^{-/-}$ (bottom). Dotted line=FMO control. n= 5-7 mice per genotype.

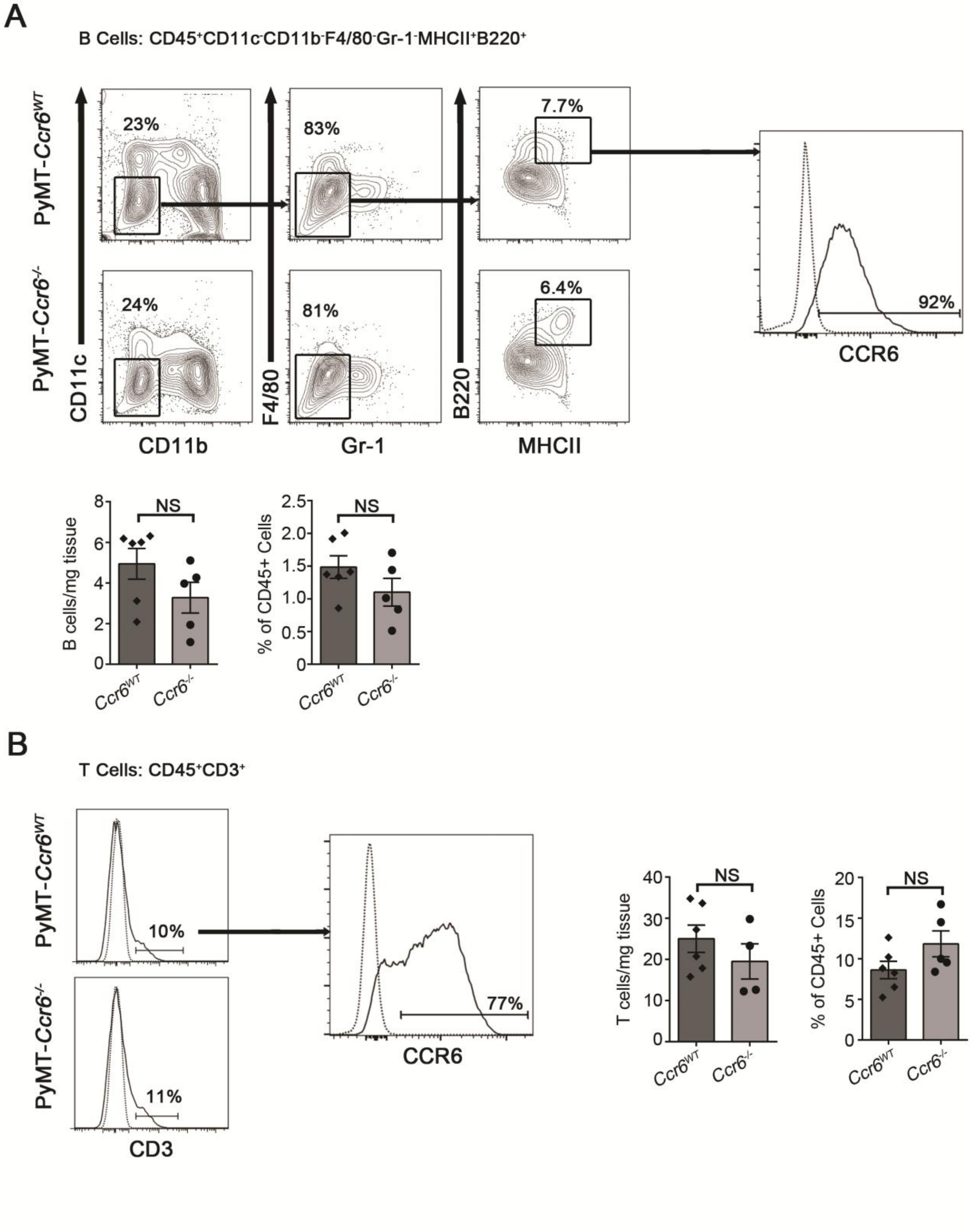

Figure S4: Infiltrating B and T immune cells in MMTV-PyMT *Ccr6*^{WT} and *Ccr6*$^{-/-}$ mammary tumors as determined by flow cytometry

All cells were gated for CD45^{+}. **(a)** MMTV-PyMT *Ccr6*^{WT} B cells were assessed for CCR6 expression (right), and proportions and numbers of B cells compared between MMTV-PyMT *Ccr6*^{WT} and *Ccr6*$^{-/-}$ (bottom). **(b)** MMTV-PyMT *Ccr6*^{WT} T cells were assessed for CCR6 expression (middle), and proportions and numbers of T cells compared between MMTV-PyMT *Ccr6*^{WT} and *Ccr6*$^{-/-}$ (right). Dotted lines=FMO controls. n= 5-7 mice per genotype.

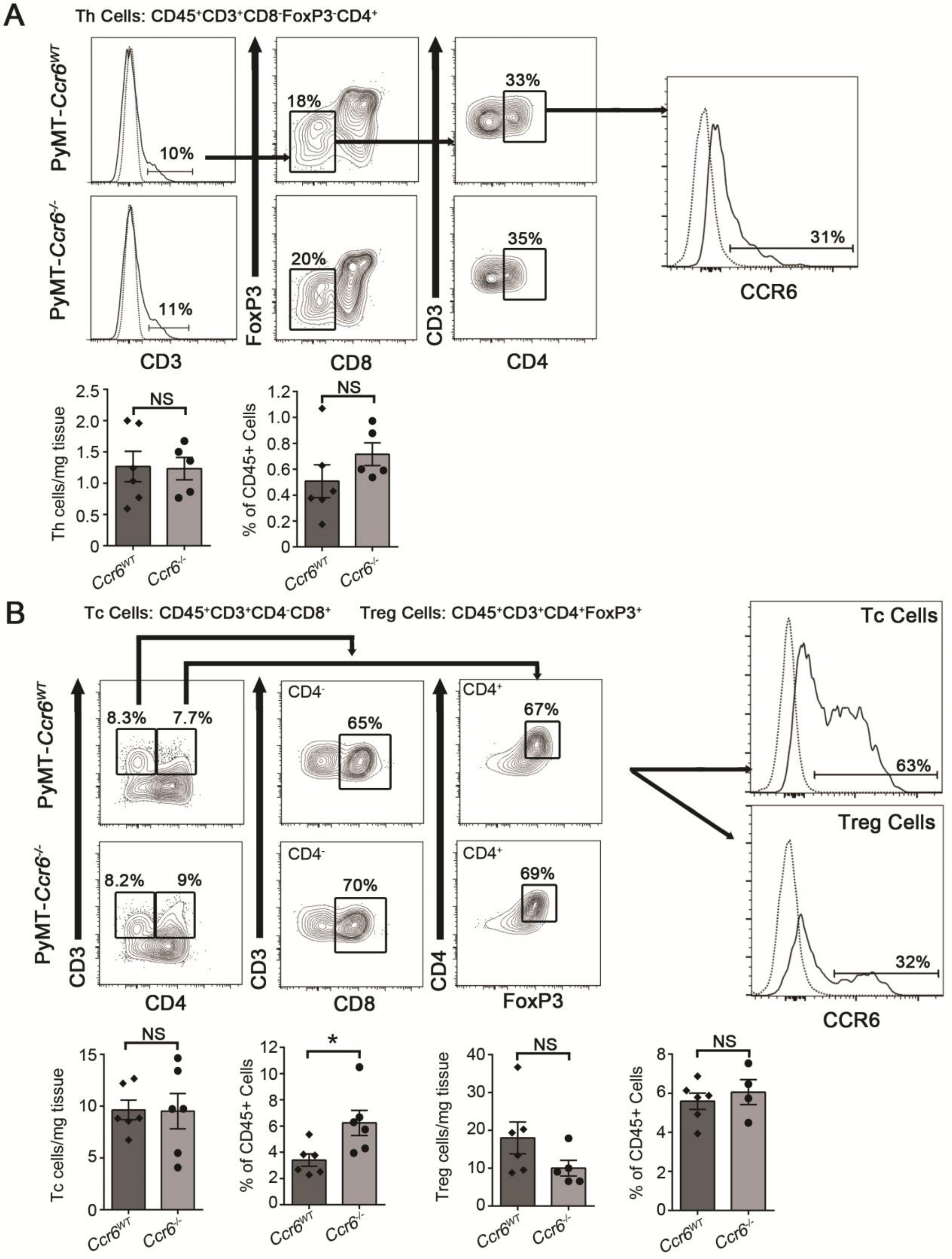

Figure S5: Infiltrating T cell subsets in MMTV-PyMT *Ccr6*^{WT} and *Ccr6*$^{-/-}$ mammary tumors as determined by flow cytometry

All cells were gated for CD45^{+}. **(a)** MMTV-PyMT *Ccr6*^{WT} helper T cells (Th) were assessed for CCR6 expression (right), and proportions and numbers of Th cells compared between MMTV-PyMT *Ccr6*^{WT} and *Ccr6*$^{-/-}$ (bottom). **(b)** MMTV-PyMT *Ccr6*^{WT} cytotoxic T cells (Tc) and regulatory T cells (Treg) were assessed for CCR6 expression (right), and proportions and numbers of Tc/Treg cells compared between MMTV-PyMT *Ccr6*^{WT} and *Ccr6*$^{-/-}$ (bottom). Dotted lines=FMO controls. n= 5-7 mice per genotype.
